# Parasomnias in Post-Secondary Students: Prevalence, Distress, and Coping Strategies

**DOI:** 10.3390/bs14080646

**Published:** 2024-07-26

**Authors:** Catherine S. Fichten, Eva Libman, Sally Bailes, Mary Jorgensen, Alice Havel, Yuxuan Qin, Laura Creti, Huanan Liao, Bianca Zlotea, Christine Vo, Jillian Budd, Abigaelle Vasseur, Tanya Pierre-Sindor, Georgiana Costin

**Affiliations:** 1 Department of Psychology, Dawson College, Montreal, QC H3Z 1A4, Canada; ahavel@dawsoncollege.qc.ca (A.H.); bianca.zlotea@yahoo.com (B.Z.); 2 Adaptech Research Network, Montreal, QC H3Z 3G4, Canada; mjorgensen07@ubishops.ca (M.J.); yuxuan.qin@mail.mcgill.ca (Y.Q.); huanan.liao@mail.mcgill.ca (H.L.); christine.vo@hyperqube.ca (C.V.); jillian.budd@mail.mcgill.ca (J.B.); abivasseur@gmail.com (A.V.); tanya.pierre-sindor@mail.mcgill.ca (T.P.-S.); georgiana.costin@mail.mcgill.ca (G.C.); 3 Behavioural Psychotherapy and Research Unit, Jewish General Hospital, Montreal, QC H3T 1E4, Canada; eva.libman@mcgill.ca (E.L.); sally.bailes@mcgill.ca (S.B.); laura.creti@mcgill.ca (L.C.); 4 Department of Psychiatry, McGill University, Montreal, QC H3A 1A1, Canada; 5 Department of Psychology, McGill University, Montreal, QC H3A 1A1, Canada; 6 Department of Education, McGill University, Montreal, QC H3A 1A1, Canada

**Keywords:** parasomnia, college student, prevalence, disturbance, coping

## Abstract

Parasomnias are a group of sleep disorders characterized by abnormal and unpleasant motor, verbal, or behavioral events that occur during sleep or during transitions between wake and sleep states. They disrupt sleep and can have a detrimental impact on the individual experiencing them. Our goal was to identify types of parasomnias and their prevalence in the current and recent post-secondary student population and to explore their coping strategies for parasomnias they found distressing. Seventy-seven post-secondary students completed the 21-item Munich Parasomnia Screening (MUPS) frequency scale. They also rated, on a 10-point scale, how disturbing each parasomnia experienced was. Not only did 92% percent of students report at least one parasomnia, but our results also indicate that the vast majority of students experienced several parasomnias. This led us to investigate the likelihood of the co-occurrence of different parasomnias. With respect to the level of subjectively experienced distress, the most prevalent parasomnias were not always the more disturbing. Coded open-ended responses about what students do about the disturbing parasomnias indicate that grounding strategies (i.e., coping strategies that help manage distressing feelings) and physical manipulation of one’s body were the most common, although most participants indicated that in spite of distress, they do nothing to cope. In conclusion, our study found a strikingly high prevalence of parasomnias in this sample of young adults and a lack of knowledge about effective means of dealing with these. Therefore, we provide some accepted ways of dealing with these.

## 1. Introduction

Parasomnias are a group of sleep disorders characterized by abnormal and unpleasant motor, verbal, or behavioral events that occur during sleep or wake to sleep transitions [1]. They can be quite distressing for the individual experiencing them and are often very disturbing for a bed partner.

### 1.1. REM and NREM Parasomnias

During sleep, the brain cycles regularly between wakefulness and two sleep phases (nonrapid eye movement (NREM) and rapid eye movement (REM)). The classification of most parasomnias depends on whether they have emerged from NREM or REM sleep [2]. The following three tables show examples of the most common of these [3].

NREM parasomnias involve physical and verbal activity of varying complexity. Typically, the sleeper returns to sleep and is amnesic in the morning (behavior is reported by others, discovered because items have been moved or used during the night, or the events have resulted in injury). During NREM parasomnias people’s eyes tend to be open. NREM parasomnias occur most frequently in the first third of the night (see Table 1).

In contrast, characteristics of REM parasomnias include verbalizations and actions consistent with dream enactment. These parasomnias often include fight or flight behaviors that usually awaken the sleeper who, unlike those with NREM parasomnias, can often recall the event, the dream, and the associated actions. During REM parasomnias people’s eyes tend to be closed. REM parasomnias occur most often in the last third of the night (see Table 2).

Other parasomnias, including exploding head syndrome, sleep enuresis (bed wetting), and sleep-related suffocating, are not specific to either NREM or REM sleep phase and may occur at any point during the sleep cycle (see Table 3).

Notably, the most frequently used parasomnia questionnaire—the Munich Parasomnia Screening Questionnaire (MUPS) [4]—includes several items that occur at night and may disrupt sleep, which are classified by the ICSD-3 [3,5] as movement disorders, such as periodic twitching and kicking while asleep, nocturnal leg cramps, and sleep-related bruxism. In addition, the MUPS also contains items that do not emerge from a specific sleep stage, but rather are more accurately defined as phenomena occurring during the transition from wake to sleep at the beginning of the night, sleep to wake at the end of the night, or from one sleep stage to another. Examples of such “transition phenomena” are hypnagogic (while falling asleep) and hypnopompic (while waking up) hallucinations, and hypnic jerks and rhythmic leg movement, which occur at the transition from quiet wakefulness to sleep. As Stieglitz and Heppner [6] noted, “Nocturnal phenomena, such as teeth grinding (bruxism), nocturnal cramps, repeated twitching of the legs or kicking, REM sleep-associated cardiac arrest or REM sleep-associated AV nodal block, as well as chocking and suffocating during sleep, are no longer classified by name in the current ICSD-3. But these other parasomnias may be found in sleep self-rating tools, such as the Munich parasomnia screening (MUPS)”.

### 1.2. Causes and Consequences

Generally, triggers and exacerbating factors for parasomnias include sleep deprivation, delayed sleep phase disorder, insomnia, and anxiety [7]. External events such as noise and temperature change, or internal events such as sleep apnea, restless leg syndrome, and periodic limb movement disorder, may cause an arousal or partial awakening, usually from NREM Stage N3 sleep [8]. These occur in the first 90 minutes of the sleep period. Arousal or partial awakening can also occur during NREM stages N1 or N2 sleep later in the night. Shift work may also increase risk for REM and non-REM parasomnias [9].

These negative sleep-related phenomena can have detrimental impacts, such as sleep-related injuries (e.g., from sleepwalking and REM behavior disorder), daytime sleepiness (e.g., sleep deprivation), and psychological distress (e.g., nightmare disorder, sleep-related hallucinations, sleep paralysis). For example, Kelly [10] found that, in a sample of 373 American undergraduates, individuals who reported frequent nightmares also reported higher levels of depression, anxiety, and neuroticism. In addition, Alshahrani et al. [11] also found that parasomnias among university students were significantly associated with depression and anxiety, corroborating research on the well-established links between parasomnias and mental health issues [9].

### 1.3. Prevalence

Past research on parasomnias predominantly focused on children [7], who typically “outgrow” these, and on clinical populations [12]. Studies targeting college students have been limited until recently.

Indeed, we have been able to find only four studies where the focus was on college students or young adults. An early study [13] in Nigeria found that over 70% of university students reported experiencing at least one parasomnia at some point in the past, with nightmares, enuresis, sleep paralysis and night terrors being the most common. A more recent study by Kirwan and Fortune [14] in Ireland investigated the one-year prevalence rates of parasomnias among 135 university students. This study showed that nearly all participants (98%) had experienced at least one parasomnia during the past year, with over a quarter (28%) reporting seven or more. Hypnic jerks, nightmares, and sleep-talking were the most prevalent. Similarly, Alshahrani et al. [11] in Saudi Arabia studied a large university sample of 1296 students and found that 81% reported at least one parasomnia. The most prevalent during the previous six months were sleep-talking (51%), nightmare disorder (50%), and confusional arousals (40%). They also reported that parasomnias among university students were significantly associated with psychological stress, depression, and anxiety disorders. In a study on young adults [10], Matsui et al. found that 2.2% of their participants experienced sleep-related eating disorder-like behavior. This parasomnia was associated with smoking, the use of hypnotic medication, and previous and/or current sleepwalking.

Although these studies show that parasomnias, especially nightmares, are common among college students, there are a variety of minor differences in prevalence among the findings. These may stem from a range of factors, such as cultural, national, or regional differences, variations in the measures, and discrepancies in the timeframe (e.g., over the past fourteen days, past six months, past year, or lifetime). Notably, there are no data on the one-year prevalence of parasomnias among post-secondary students in North American countries, including Canada.

None of these investigations explored how distressing the parasomnias were to students nor how students dealt with these. In addition, there has been no systematic investigation of co-occurrence of the parasomnias in the same individual.

### 1.4. Present Study

The goals of this study were to (1) explore the one-year prevalence of the 21 parasomnias listed on the most frequently used, validated parasomnia measure in a population of current and recent post-secondary students, (2) evaluate whether an individual with one specific parasomnia will have another specific parasomnia, (3) assess how disturbing each parasomnia was to current and recent students, and (4) explore what they did to cope with the parasomnia.

We hypothesized that, as in other studies:

(1) Over 70% of students would report at least one parasomnia during the past year;
(2) Most students would report two or more types of parasomnias;
(3) Nightmare disorder would be the most prevalent parasomnia reported.

In addition, the following hitherto uninvestigated areas were explored. These additional analyses add new, interesting, and useful insights:

(4) Investigating students’ distress levels related to specific parasomnias;
(5) Identifying their coping strategies for each parasomnia;
(6) Exploring the co-occurrence of different parasomnias in this population.

## 2. Materials and Methods

### 2.1. Participants

The participants consisted of 77 individuals, 52 current and 25 recent (had been students in the past five years) Canadian post-secondary students. We excluded those who indicated having a disability from the sample. This was, in part, because individuals with certain disabilities (e.g., psychological disabilities such as anxiety and depression) are more likely to experience particular parasomnias such as nightmares and sleep-waling [11]. Forty-seven students were female, twenty-nine were male, and one indicated having a non-binary gender. The median age of the sample was 22 (range 18–31).

### 2.2. Measures

#### 2.2.1. Demographics

We asked participants to indicate their gender (textbox), age, the presence or absence of a wide variety of disabilities (e.g., attention deficit hyperactivity disorder, specific learning disorder, mobility impairment, sensory disability) including psychological disorders, and whether they were currently or recently (past five years) a post-secondary student.

#### 2.2.2. Munich Parasomnia Screening Questionnaire (MUPS)

This 21-item measure evaluates the frequency of experiencing 21 parasomnias (see Appendix A). Because we were interested in a one-year prevalence, we modified the MUPS frequency scoring to a 6-point Likert-type scale (1 = never, 2 = very rarely, 3 = rarely, 4 = sometimes, 5 = often, 6 = very often). Fulda et al. [4] reported good validity for this measure. Heinzer and his graduate students (Raphael Heinzer, 26 June 2023, personal communication [15]) used a French language version. Since Heinzer indicated that this was not a validated version, we made changes to reflect French language usage in one of Canada’s largest provinces: Québec. It is important to note that the MUPS categories do not consistently reflect the current ICDS-3 [3,5] classification.

For each MUPS item we also asked participants to indicate how disturbing they found the phenomenon. We identified the level of disturbance using a 10-point scale (1 = not at all disturbing to 10 = very disturbing). For all items participants rated as occurring at least rarely (i.e., =>3 on the MUPS frequency scale), we asked them to write what they did to cope with and manage these. 

### 2.3. Procedure

We conducted a bilingual (English, French) online survey between October and December 2023 [16]; although participants hailed from all of Canada’s ten provinces, in one province, Quebec, the language of the majority is French. The host institution’s Research Ethics Board approved the study (Certificate: FICHC23244335). Participant recruitment proceeded in the following ways: (1) email invitations were sent to current and former Canadian postsecondary students who had participated in our previous research and had indicated that we could contact them for future studies, (2) announcements were emailed to discussion lists focusing on Canadian postsecondary education, and (3) student team members helped by recruiting friends and acquaintances. All students were participating in a larger investigation, and everyone who completed the survey received a $30 (Canadian) Amazon gift card. 

## 3. Results

### 3.1. One Year Prevalence

In accordance with Hypothesis 1, the one-year prevalence data show that 92% of participants experienced at least one parasomnia during the past year. As Table 4 shows, our prevalence scores are generally somewhat lower than those of Kirwan and Fortune [14], although the trends are the same, with a high correlation of *r*(19) = 91, *p* < 01 between the prevalence scores in the two studies.

As predicted in Hypothesis 3, nightmares were most prevalent. Table 4 also shows that other common parasomnias, in rank order, were hypnic jerks, sleep talking, sleep-related bruxism, nocturnal leg cramps, periodic twitching and kicking while asleep, and rhythmic leg movements while falling asleep.

### 3.2. Number of Parasomnias Experienced

**Figure 1:**
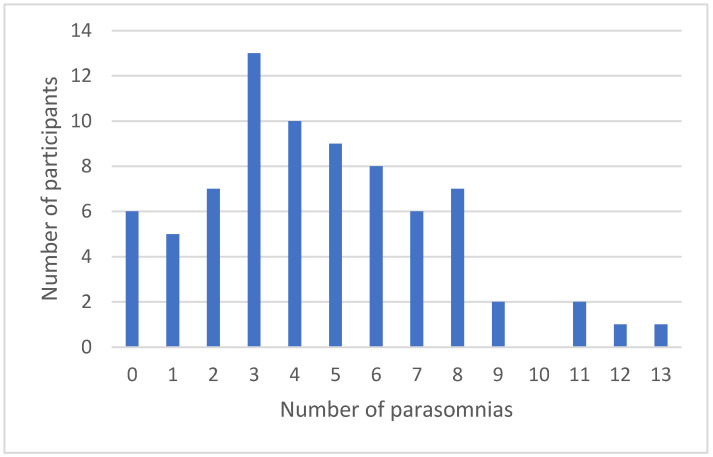
Number of parasomnias experienced per participant.

Consistent with Hypothesis 2, most participants experienced several different parasomnias. Among those who experienced at least one parasomnia (i.e., 92% of the sample), the mean number experienced was five (SD = 3, median = 4, range = 1 to 13). The results also show that 93% (i.e., 66/71) of those who have at least one parasomnia are likely to have at least one additional parasomnia. Figure 1 shows the number of students experiencing between 0 and 13 parasomnias.

### 3.3. How Disturbing Is Each Parasomnia?

Table 5 shows that among those who experience parasomnias, the most frequently occurring parasomnias (i.e., very rarely to very often) are nocturnal leg cramps and hypnagogic/hypnopompic hallucinations. Moreover, Table 5 also shows that the most common parasomnias are not necessarily the most disturbing.

Although we assessed how disturbing each parasomnia was to students, as we noted in item (4) we had no hypotheses related to distress. When we investigated, we found that the most disturbing/distressing parasomnias, in rank order, were sleep enuresis, sleep paralysis, sleep terrors, sleep-related abnormal choking/suffocating, nocturnal leg cramps, nightmares, and exploding head syndrome. When we took into account only those parasomnias that participants indicated occurred often or very often during the year (i.e., frequency score of 5 or 6), nocturnal leg cramps, sleep enuresis, sleep paralysis, nightmares, and nocturnal eating were the most disturbing.

### 3.4. Coping Strategies

As noted in Question (5), we explored what participants did to cope with the most disturbing parasomnias they experienced. To do so, we developed a coding manual to categorize participant responses [17]. There were two coding teams, each comprised of two people. Coders were trained to a minimum of 70% inter-rater agreement; mean coding agreement attained by the two teams was 89%. In the case of discrepancies, coders discussed their responses and agreed upon a consensus code. Examples are available in Table 6.

After coding the responses, we analyzed the frequencies of responses for each of the coding categories for each parasomnia. Table 7 shows that most participants said that they did nothing to manage their parasomnia or did not know how to cope with their parasomnia. This is particularly true for parasomnias that participants did not report as disturbing, such as sleep-related groaning, sleepwalking, sleep talking, and rhythmic leg movements while falling asleep.

Table 7 also shows that grounding strategies, which are used to cope with distressing feelings, were the most frequently mentioned coping strategies. The only parasomnias for which participants did not mention using grounding strategies were those that they did not find especially disruptive or that they found to be interesting or funny (e.g., sleep talking). The second most frequently mentioned was a preventative strategy, which participants with several different parasomnias reported using. Table 7 shows that half of the participants who implemented physical manipulation of the body reported experiencing nocturnal leg cramps. It is also important to note that a very large number of participants, despite finding the parasomnia disruptive, did nothing. In fact, doing nothing was the most popular strategy reported.

### 3.5. Which Parasomnias Tend to Occur Together?

We were also interested in exploring the co-occurrence of parasomnias. Our findings in Appendix B indicate the relative likelihood of a participant having two specific parasomnias. We used the following formula to calculate co-occurrence:

$$\mathrm{Co}\text{-}\mathrm{occurrence}\;\mathrm{Relative}\;\mathrm{Frequency}=\frac{\mathrm{Number}\;\mathrm{of}\;\mathrm{times}\;\mathrm{two}\;\mathrm{events}/\mathrm{items}\;\mathrm{occur}\;\mathrm{together}}{\mathrm{Total}\;\mathrm{number}\;\mathrm{of}\;\mathrm{occurrences}\;\mathrm{of}\;\mathrm{one}\;\mathrm{or}\;\mathrm{both}\;\mathrm{events}/\mathrm{items}}$$

Appendix B shows the percentage of participants who reported at least two specific parasomnias (i.e., if a participant had one or the other parasomnia, the percentage reported is the likelihood that they had both parasomnias). For example, the co-occurrence relative frequency of hypnic jerks and nightmares is 58%, indicating that if a participant has either one of those parasomnias, the probability of having the other is 58%. The benefit of using relative frequency is that it is largely independent of the individual frequency of each parasomnia; it accounts mainly for co-occurrence when both appear. Thus, if the individual frequency of a parasomnia is very low, this method of calculation will affect the co-occurrence only minimally. Few parasomnias have a co-occurrence relative frequency greater than 30% (indicated in bold in Appendix B).

How different parasomnias co-occur is not intuitively obvious, with REM and NREM parasomnias co-occurring with each other. This lack of predictable patterns is substantiated by the factor analysis conducted by Kirwan and Fortune [14] on students’ lifetime prevalence data; this shows that the factors do not group NREM and REM parasomnias separately as does a factor analysis conducted by our team on one-year prevalence data.

## 4. Discussion

Our findings in this under-researched population show that the one-year prevalence of parasomnias among North American college students and young adults is very high and that many of these parasomnias are disturbing and distressing, with nightmares being especially common. This is true even though our sample was primarily healthy since we excluded those who self-reported a disability, including mental health problems (cf. [11]). In this mainly healthy sample, we found the number of students who experienced parasomnia and negative sleep experiences at least once during the past year to be extraordinary. Moreover, our findings show that when a student has a parasomnia, they are likely to have other parasomnias as well; very few students have just one.

As predicted in Hypothesis 1, 92% of our sample had at least one parasomnia. This was consistent with the results of Kirwan and Fortune [14], who reported that 98% of their sample experienced one or more recent or current parasomnias. As predicted in Hypothesis 2, most (77%) participants had at least three. Having only one parasomnia was rare. As predicted in Hypothesis 3, nightmares were particularly common, with 82% of participants reporting that they had experienced these during the past year. This was also true in the three other studies investigating parasomnias in college students [11,13,14]. In the present sample, parasomnias experienced were generated from all stages and phases of sleep, including transitions between wake and sleep states.

In general, sleep deprivation, delayed sleep phase disorder, insomnia, and anxiety [7], noise and temperature change, as well as conditions such as sleep apnea, restless leg syndrome, and periodic limb movement disorder can have detrimental impacts and cause a variety of parasomnias [8].

### 4.1. Relative Co-Occurrence

We wanted to learn which parasomnias exist together. Our findings indicate that nightmares and sleep talking have the greatest overlap with other parasomnias. Others seem to overlap minimally (e.g., exploding head syndrome, sleep terrors). Knowing how likely parasomnias co-occur demonstrates that REM and NREM parasomnias did not appear to follow any predictable or expected principle.

The results show that participants who had nightmares (a REM parasomnia) were also likely to have the following NREM parasomnias (hypnic jerks and sleep talking), as well as parasomnias that can occur in either REM or NREM stages, such as nocturnal leg cramps and sleep-related bruxism. Those who experienced sleep talking (an NREM parasomnia) were also likely to have nightmares (a REM parasomnia), as well as hypnic jerks and periodic twitching and kicking while asleep (NREM parasomnias). Many other parasomnias were also associated with each other.

### 4.2. Parasomnia Frequency, Disturbance and Distress Aspects

Our research examined not only the prevalence, but also the one-year frequency and the degree of disturbance of parasomnias. Eliciting subjective perceptions about how disturbing the phenomenon is merits attention, since the parasomnia that causes the most distress for an individual is not necessarily the most frequently experienced one. The most common prevalence of parasomnias, in rank order, were nightmares, hypnic jerks, sleep talking, sleep-related bruxism, nocturnal leg cramps, rhythmic leg movements while falling asleep, and periodic twitching and kicking while asleep.

Our findings also show that although few participants reported nocturnal leg cramps and hypnagogic/hypnopompic hallucinations, these occurred more often than other parasomnias. Both these parasomnias were perceived as moderately disturbing. In addition, while many participants experienced nightmares as well as sleep related bruxism, these were generally not experienced frequently. However, for the many individuals who did experience them, these parasomnias were quite disturbing. When we examined only those parasomnias that participants indicated occurred often, nocturnal leg cramps, sleep enuresis, sleep paralysis, nightmares, and nocturnal eating were the most disturbing.

There were several other disturbing parasomnias, but these were experienced by few participants or were experienced infrequently. With few exceptions, disturbing parasomnias tended to occur during NREM and in those which involved physical movements. In rank order these are sleep enuresis, sleep paralysis, sleep terrors, sleep-related abnormal choking/suffocating, nocturnal leg cramps, nightmares, and exploding head syndrome.

It is important to note that some of the parasomnias are unique to age groups. For example, sleep enuresis is common among children [18] and REM sleep behavior disorder is more common among older adults [19], not among the young adults in our sample.

### 4.3. How Do Students Cope with Disturbing/Distressing Parasomnias?

It is important to note that the most common way that students managed their parasomnia, be it frequent or infrequent and be it very or mildly disturbing, was to do nothing about it. This was especially true of hypnic jerks, nightmares, nocturnal leg cramps, and sleep related bruxism. Even for disturbing parasomnias, such as sleep related groaning, students reported that they did nothing to cope with it, suggesting that they did not know what to do. It is worth mentioning that students did not report that they turned to self-medication with drugs or alcohol.

The largest number of students who indicated a means of trying to manage their parasomnia used a grounding strategy (e.g., taking actions to relax and calm oneself down). Participants implemented grounding for many parasomnias, including nightmares, exploding head syndrome, hypnagogic hallucinations, and confusional arousals, among others. It is important to note that many of those reporting nightmares also reported using distraction strategies such as scrolling on their smartphones or texting with friends. 

The second most frequently mentioned coping strategy was prevention. Students were especially likely to use these strategies for nightmares (e.g., changing what they chose to watch or listen to before bed, changing their bedtime) and tooth grinding (e.g., using jaw exercises or mouth guards). Students tended to use physical manipulation of their body for physical parasomnias such as nocturnal leg cramps, rhythmic movement disorder, and sleep related abnormal choking suffocating.

### 4.4. Developmental and Age-Related Considerations

Notably, some parasomnias are more characteristic in different age groups. For example, sleep enuresis is relatively common among children [18,20], but very infrequent in adults; REM sleep behavior disorder usually only begins in the fifth or sixth decade of life [19,20], suggesting a developmental component in this sleep-related experience. Our own study, by its unique focus on parasomnias and other sleep disturbances in the late adolescence and early adulthood college student cohort, adds a hitherto unexplored piece to general knowledge. It is known that the various parasomnias follow somewhat predictable patterns across the lifespan. The present data appear to reinforce the existence of a transitional developmental trajectory between childhood and later adulthood, which includes parasomnias and other sleep disturbances most associated with these life stages.

It has been noted that many parasomnias, particularly those associated with NREM sleep, have the highest prevalence in children, tend to decrease in frequency after the onset of puberty, and become rare in adulthood. This pattern suggests a developmental and maturational trajectory of the nervous system (e.g., night terrors) as well as the development of self-regulation of body functions, even while asleep (e.g., enuresis) [21].

Nevertheless, sleep terrors were reported by a surprising number of participants (17%) overall, while 6.5% rated them as very frequent and disturbing. Night terrors have a prevalence of 37% to 20% during early childhood, and a prevalence of 2.2% in the general adult population [22].

The experience of occasional nightmares is very common in the general adult population, ranging from 22% to 45% [23], and in children, ranging from 60% to 75% [7]. Nightmare disorder, the diagnosis of which is made largely by self-report, occurs in about 5% of children [22] and in about 4% of the general adult population [24]. In the present young adult sample, 82% reported the occurrence of nightmares in the past year, and 6.5% rated both the frequency and disturbance level as high. It is important to take into account the unique pressures experienced by young adults as they advance through their education and assume careers and other responsibilities.

## 5. Limitations

There are issues related to the generalizability of the findings. The frequency of occurrence of these negative sleep events appears, in general, very high for a young, relatively healthy sample. This may in part be due to the fact that they are self-reported compared to some research estimates that are based on diagnostic interviews or objective measurements such as polysomnography. Some parasomnias such as sleep terrors and REM sleep behavior disorder may be under-reported by our sample because these need corroboration from someone sleeping in proximity to the student. Furthermore, we excluded students with mental health related disabilities, even though we expected that students with various disabilities would have higher rates of parasomnias. Research on students with disabilities and parasomnias, as well as on other risk factors and resilience, are ongoing in our laboratory. In addition, it was not possible to calculate power for basic frequency and coding data and the sample size of 77 is relatively small. Also, there were few participants who indicated a non-binary gender (e.g., transgender, gay, lesbian, agender), and most participants studied in only one of Canada’s ten provinces.

## 6. Conclusions and Implications

Our study brings new insights into clinical monitoring by highlighting the extensive prevalence and co-occurrence of specific parasomnias among current and recent students. Our findings clearly show that although 92% of students experience an assortment of parasomnias, with a large variety of frequency and levels of disturbance, they know little about effective means of managing these. In particular, raising awareness of nightmares as an important health concern is critical. In a study of 747 undergraduate students, only 11% of participants with significant nightmares reported having told a healthcare provider about their nightmares [25].

Moreover, in our team’s sleep clinic, those presenting with sleep problems rarely mention parasomnias. The same experience is true of general practitioners and medical sleep specialists, as our Advisory Board members indicated [26]. Our study highlights the need for comprehensive sleep assessments and targeted interventions. Clearly it is important to have an idea about techniques to address the most disturbing and the most frequent parasomnias. Regrettably, there are very few “tried and true” validated means of dealing with these (see current suggestions for the most common or distressing parasomnias in Appendix B).

A more salient point is how to communicate potential strategies to those individuals who experience high levels of disturbance, especially to students and other young adults. They do not appear to seek professional help, as many prefer peer advice, nor are they likely to read professional journals. The answer appears to lie in social media platforms such as Redditt, TikTok and Instagram. Perhaps these will become the new vehicles of dissemination of research-based strategies to diminish the occurrence of disturbing parasomnias in postsecondary students.

## Figures and Tables

**Table 1:** Common disorders of arousal from NREM sleep.

Name of Parasomnia	Description
Confusional arousals	Partial awakenings during slow-wave (stage 3) sleep. The individual is confused, disoriented to time and space.
Sleepwalking	Ambulatory behavior during slow wave (stage 3) sleep.
Sleep-related eating	Recurrent episodes of involuntary binge eating after partial awakening from sleep.
Sleep talking	Speaking aloud during slow wave (stage 3) sleep.
Sleep terrors	Episodes of intense fear, accompanied by increased autonomic nervous system activity.

**Table 2:** Common disorders of arousal from REM sleep.

Name of Parasomnia	Description
Nightmares	Repeated occurrence of frightening dreams that lead to awakening.
REM sleep behavior disorder	Vivid dreaming (often frightening) without the usual accompanying reduced muscle tone that results in behavioral acting out of the dream content.
Sleep paralysis	REM-based reduced skeletal muscle tone perseverates into wakefulness, resulting in the inability of the affected person to move or speak during wakefulness.

**Table 3:** Common disorders of arousal emerging from either sleep phase.

Name of Parasomnia	Description
Exploding head syndrome	Sudden loud noise in the head that occurs during wake-sleep transitions or while awakening in the middle of the night.
Sleep enuresis	Involuntary release of urine while sleeping.
Sleep-related abnormal choking/suffocating	Swallowing with aspiration during sleep.

**Table 4:** Frequency of MUPS items.

^1^ Parasomnia Label	^2^ REM/NREM	^3^ Our Data: % with the Parasomnialiwenn = 77	^3^ Kirwan & Fortune [14] % with the Parasomnia
Confusional arousals	NREM	19%	**30%**
Exploding head syndrome	Either	16%	16%
Hypnagogic/hypnopompic hallucinations	Transition	21%	**41%**
Hypnic jerks	NREM	**53%**	**79%**
Nightmares	REM	**82%**	**74%**
Nocturnal eating	NREM	18%	**33%**
Nocturnal leg cramps	Either	**32%**	**44%**
Periodic twitching and kicking while asleep	NREM	**25%**	**28%**
REM sleep behavior disorder	REM	14%	16%
Rhythmic leg movements while falling asleep	Transition	**25%**	**38%**
Rhythmic movement disorder	NREM	13%	19%
Sleep enuresis	Either	5%	2%
Sleep paralysis	REM	14%	15%
Sleep-related abnormal choking/suffocating	NREM	5%	10%
Sleep-related bruxism	Either	**34%**	**27%**
Sleep-related eating	NREM	0%	2%
Sleep-related groaning	Either	17%	**26%**
Sleep talking	NREM	**38%**	**58%**
Sleep terrors	NREM	17%	**30%**
Sleep walking	NREM	4%	9%
Violent behavior	NREM	8%	11%

^1^ See Appendix A for MUPS questionnaire items related to parasomnia labels. ^2^ “REM/NREM” defines the sleep phase in which the parasomnia occurs. “Transition” refers to transition between sleep stages and “either” suggests that the parasomnia can occur during either REM or NREM. ^3^ Parasomnias with over 25% prevalence are bolded.

**Table 5:** Frequency and disturbance of MUPS items.

Parasomnia Label	Number of Participants Reporting Parasomnia	^1^ How Often Do You Have This Parasomnia (Mean: Very Rarely = 2 to Very Often = 6)	How Disturbing/Distressing Was This?	
			^2^ Number of Participants	^3^ Mean Disturbance 1–10
Confusional arousals	15	3	9	3.56
Exploding head syndrome	12	3	6	**5.50**
Hypnagogic/hypnopompic hallucinations	16	**4**	5	4.80
Hypnic jerks	41	3	26	3.42
Nightmares	63	3	41	**5.56**
Nocturnal eating	14	3	7	4.14
Nocturnal leg cramps	25	**4**	14	**6.50**
Periodic twitching and kicking while asleep	19	3	11	2.82
REM sleep behavior disorder	11	2	4	5.25
Rhythmic leg movements while falling asleep	19	3	10	3.00
Rhythmic movement disorder	10	3	7	1.86
Sleep enuresis	4	3	1	**8.00**
Sleep paralysis	11	3	6	**7.50**
Sleep-related abnormal choking/suffocating	4	3	3	**7.33**
Sleep-related bruxism	26	3	18	4.22
Sleep-related eating	0	0	0	0.00
Sleep-related groaning	13	3	5	1.40
Sleep talking	29	3	13	1.69
Sleep terrors	13	3	5	**7.40**
Sleepwalking	3	3	2	1.50
Violent behavior	6	3	2	2.00

^1^ 2 = very rarely, 3 = rarely, 4 = sometimes, 5 = often, 6 = very often. Scores = > 4 are bolded. ^2^ Number of participants who had the parasomnia, excluding very rarely = 2. ^3^ How disturbing/distressing was this? 1= not at all, 10 = very disturbing. Scores => 5.5 are bolded.

**Table 6:** Examples of coding categories and responses.

Categories	Examples of Codes
Preventative interventions	“Changed the type of content I was looking at before going to sleep.” “Put physical barriers (i.e., cushions or pillows) to prevent me from falling from my bed.”
Distraction/shifting focus	“Listen/watch videos to help me feel better.” “I will text my friends.”
Grounding strategies	“Take a minute before going back to sleep–breathe, drink some water, calm down.” “So I just try to stay calm in my mind until I muster enough energy to force my body to wake up.”
Physical manipulation of body/exercise	“Moved my legs for several minutes until I felt circulation.” “I will straighten my calf and pull my big toe hard.”
Other strategies	“I tried to think why I was having unsettling dreams and made a cognitive effort to understand my feelings that were not fully in my conscious awareness.” “I sometimes share my dreams with family or friends.”

**Table 7:** How participants coped with parasomnias.

	Coping Responses by a Participant						
^1^ Parasomnia Labels	Preventative Interventions	Distraction/Shifting Focus	Grounding Strategies	Physical Manipulation of Body/Exercise	Other Strategies	^2^ Total Number of Participants Using These Coping Responses	^3^ Reported They Did Nothing
Confusional arousals	1		4			5	
**Exploding head syndrome**	1	1	2			4	1
Hypnagogic/hypnopompic hallucinations			1			1	3
Hypnic jerks			2	1		3	16
**Nightmares**	4	5	6		4	19	11
Nocturnal eating	2		4		2	8	2
**Nocturnal leg cramps**				6		6	4
Periodic twitching and kicking while asleep					1	1	4
REM sleep behavior disorder			1			1	1
Rhythmic leg movements while falling asleep						0	3
Rhythmic movement disorder		1		1		2	2
**^4^ Sleep enuresis**							
**Sleep paralysis**	3		2	1		6	2
**Sleep-related abnormal choking/suffocating**				1		1	
**Sleep-related bruxism**	7			2	3	12	6
^4^ Sleep-related eating							
**Sleep-related groaning**						0	1
Sleep talking						0	4
**Sleep terrors**		1	1			2	
Sleep walking						0	1
Violent behavior	1					1	
Total number of codes	19	8	23	12	10	72	61

^1^ Disturbing parasomnias are bolded. ^2^ Number of participants mentioning the coping response. ^3^ Number of participants who indicated that they did nothing. ^4^ No responses were provided.

**Figure A1:**
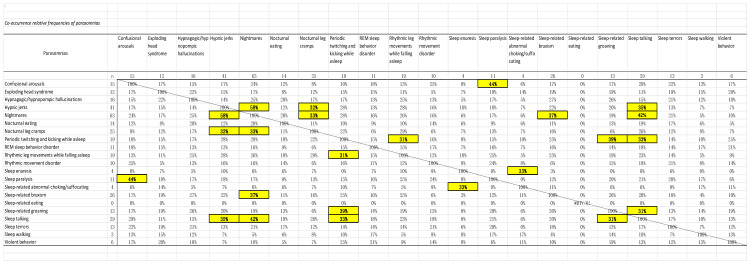
Co-Occurrence Relative Frequencies of Parasomnias. Highlighted items are those that occur at least 30% of the time.

## Data Availability

The original contributions presented in the study are included in the article, further inquiries can be directed to the corresponding author.

## Appendix A

**Table A1:** Parasomnia Labels and Corresponding MUPS Items.

Parasomnia Label in Alphabetical Order	MUPS Questionnaire Items and Order of Presentation
Confusional arousals	17. Upon waking, completely confused/difficult orientation/slowed speech
Exploding head syndrome	4. When falling asleep or waking, perceiving a loud bang, a sound similar to a bang (e.g., door bang), or having the sensation of an “explosion in the head.”
Hypnagogic/hypnopompic hallucinations	5. Auditory or visual illusions that accompany falling asleep or waking in a distressing or threatening manner (e.g., hearing sounds or voices, or seeing people or things that are not in the room)
Hypnic jerks	1. Leg or body twitches that occur suddenly and unintentionally when falling asleep, often with the sensation of falling
Nightmares	13. Frightening dreams and nightmares
Nocturnal eating	15. Waking again after falling asleep in order to eat something
Nocturnal leg cramps	7. Night-time cramps in the calves
Periodic twitching and kicking while asleep * Periodic leg movements	6. Repeated involuntary twitching of the legs or kicking during sleep (can only be observed by another person)
REM sleep behavior disorder	21. Have you ever actually done what you dreamt (e.g., gesticulating or lashing about)?
Rhythmic leg movements while falling asleep	2. While falling asleep or while half asleep, rhythmic and rapid leg movements that can also occur intentionally
* Rhythmic feet movements	
Rhythmic movement disorder	3. Rhythmic and repeated movements of the head or body when falling asleep or during night-time wake periods, e.g., nodding, rocking or swaying yourself to sleep
Sleep enuresis	12. Wetting oneself during sleep
Sleep paralysis	18. Waking with a paralysis of the whole body (not including eyes and breathing) that can last for several seconds
Sleep-related abnormal choking/suffocating	10. Choking or suffocating during sleep, or waking with the feeling of choking or suffocating
* Sleep-related abnormal swallowing	
Sleep-related bruxism	8. Teeth grinding during the night
Sleep-related eating	16. While asleep, eating something or preparing a meal that can have unusual or inedible ingredients (e.g., ice-cream and cheese, washing-up liquid instead of butter)
Sleep-related groaning	11. Loud and repeated sighing and moaning during sleep
Sleep talking	9. Talking during sleep
Sleep terrors	14. Waking with severe anxiety and, possibly, screaming, with no recollection of a dream
Sleep walking	19. Sleepwalking or sitting up while still asleep without leaving the bed
Violent behavior	20. Have you ever thrashed around, hitting or kicking?

* MUPS item labels used by Fulda et al. (2008) [4] and Kirwan & Fortune (2021) [14] have been changed to more accurately reflect the questionnaire item.

## Appendix C

The overall recommendation is to first find out about and address underlying causes. If this does not bring about a good night’s sleep, we review therapies for the 12 most common and or distressing parasomnias below in alphabetic order. Some of these recommendations are empirically based and others are used in major hospitals (e.g., Cleveland Clinic) or by the Sleep Foundation.

**Exploding Head Syndrome.** There are no well-documented treatments. Suggestions based on case studies include education and reassurance, anxiety management, a variety of antidepressant medications and single-pulse transcranial magnetic stimulation [27,28,29].

**Hypnic Jerks.** Reassurance and education [30], clonazepam and a variety of antidepressants [31], and avoiding caffeine, exercise before sleep, emotional stress, and sleep deprivation [32] have been described as treatment options for hypnic jerks.

**Nightmares.** Imagery Rehearsal Therapy and related treatments like exposure, relaxation, and rescripting therapy are the most effective treatments [33]. Gieselmann et al. [34] also suggested Imagery Rehearsal Therapy along with lucid dreaming.

**Nocturnal Eating.** Cho et al.’s [35] review makes the following suggestions for this under-studied parasomnia: pharmacological (serotonergic) agents, psychological interventions (cognitive behavioral therapy) and interventions targeting chronobiological and psychological aspects, such as mood and stress.

**Nocturnal Leg Cramp.** For this parasomnia, recommendations include: stretching during the day and before bed, drinking plenty of water, moving around during the day, wearing comfortable shoes, and sleeping under loose covers–especially if you sleep on your back. To treat leg cramps, solutions include: grab your toes and pull them toward you, apply ice, massage the muscle, take a warm bath, stretch the muscle, get out of bed, and stand with your foot flat on the floor and press down firmly [36].

**Periodic Twitching and Kicking While Asleep.** Studies evaluating treatment are scarce and useful medications have not been sufficiently investigated [23].

**Sleep Enuresis.** Avoiding caffeine and sedatives, regular voiding every two hours, and night time alarm systems are recommended for sleep enuresis. Other solutions include Adapted Dry Behavioral Therapy (a cognitive behavioral and prompted voiding therapy that includes close observation during sleep, waking up frequently during the night (every hour), alarm use, and day-time timed voiding (effective mainly for children)) and certain medications: desmopressin and anticholinergics [37].

**Sleep Paralysis.** People who experience sleep paralysis routinely report that focusing on making small body movements, such as moving one finger, then another, helps them to recover more quickly [38]; other options include insomnia treatment and reassurance [39].

**Sleep Talking.** Recommendations to treat this parasomnia include the following: keep a consistent sleep schedule every day, avoid caffeine or other stimulants late in the afternoon and evening, give yourself time to wind down and relax for at least a half-hour before bedtime, get regular exposure to daylight, find time for physical activity during the day, create a distraction-free sleep space that has limited light or sound pollution [40].

**Sleep Terrors.** Better sleep habits, stress management, scheduled awakenings, creating a safe environment so that it poses minimal danger if such an episode occurs (e.g., closing and/or locking windows and doors, removing tripping hazards, making sure fragile objects are not in reach) [41], and antidepressants [42] are all treatments to deal with sleep terrors.

**Sleep-Related Abnormal Choking/Suffocating.** Recommendations for this parasomnia include: sleep with your head propped up so that saliva can flow down the throat, sleep on your side instead of your back, raise the head of your bed by a few inches to keep stomach acid in your stomach, and take over-the-counter medication at the first sign of a cold, allergies, or sinus problems [43].

**Sleep-Related Bruxism.** Mouth exercises, massage, mouthguards, practicing stress reduction, and medication (e.g., Botox) are recommended for sleep-related bruxism, as are the following: avoid hard foods like nuts, popcorn, and hard candies, be cautious with peanut butter and other sticky foods, don’t chew gum, adjust your sleeping position or pillow for additional head and neck support, and hot compress or an ice pack to soothe pain [44].
